# Supplementary material for: Modeling glioblastoma heterogeneity as a dynamic network of cell states
Source: Mol Syst Biol. 2021 Sep 16;17(9):e10105. doi: 10.15252/msb.202010105 (PMC8444284; doi:10.15252/msb.202010105)
Supplement: Supplementary file 5 — Source Data for Figure 3 [file MSB-17-e10105-s001.zip › Figure3A_sourcedata/GSEA_3065/hallmarks_state6.GseaPreranked.1623416308702/HALLMARK_BILE_ACID_METABOLISM.html]

Details for gene set HALLMARK\_BILE\_ACID\_METABOLISM[GSEA]

|  || Dataset | state6 |
| Phenotype | NoPhenotypeAvailable |
| Upregulated in class | na\_neg |
| GeneSet | HALLMARK\_BILE\_ACID\_METABOLISM |
| Enrichment Score (ES) | -0.3320317 |
| Normalized Enrichment Score (NES) | -1.170242 |
| Nominal p-value | 0.20401338 |
| FDR q-value | 0.3127449 |
| FWER p-Value | 0.996 |
Table: GSEA Results Summary

  

Fig 1: Enrichment plot: HALLMARK\_BILE\_ACID\_METABOLISM      
 Profile of the Running ES Score & Positions of GeneSet Members on the Rank Ordered List

  

| PROBE | GENE SYMBOL | GENE\_TITLE | RANK IN GENE LIST | RANK METRIC SCORE | RUNNING ES | CORE ENRICHMENT || 1 | FDXR |  |  | 56 | 0.241 | 0.0774 | No |
| 2 | PRDX5 |  |  | 729 | 0.103 | 0.0365 | No |
| 3 | RBP1 |  |  | 788 | 0.099 | 0.0643 | No |
| 4 | SCP2 |  |  | 1067 | 0.082 | 0.0610 | No |
| 5 | ALDH9A1 |  |  | 1537 | 0.063 | 0.0291 | No |
| 6 | PEX19 |  |  | 1815 | 0.053 | 0.0158 | No |
| 7 | CROT |  |  | 1871 | 0.051 | 0.0274 | No |
| 8 | ISOC1 |  |  | 2024 | 0.047 | 0.0264 | No |
| 9 | RXRA |  |  | 2249 | 0.040 | 0.0147 | No |
| 10 | IDH1 |  |  | 2508 | 0.033 | -0.0033 | No |
| 11 | PNPLA8 |  |  | 2796 | 0.027 | -0.0268 | No |
| 12 | SLC27A5 |  |  | 3095 | 0.020 | -0.0539 | No |
| 13 | ACSL1 |  |  | 3113 | 0.020 | -0.0491 | No |
| 14 | ABCA1 |  |  | 3161 | 0.019 | -0.0480 | No |
| 15 | SLC23A2 |  |  | 3562 | 0.010 | -0.0902 | No |
| 16 | OPTN |  |  | 3659 | 0.008 | -0.0985 | No |
| 17 | HACL1 |  |  | 3750 | 0.006 | -0.1068 | No |
| 18 | CAT |  |  | 3983 | 0.001 | -0.1331 | No |
| 19 | PECR |  |  | 4079 | -0.001 | -0.1437 | No |
| 20 | ABCD3 |  |  | 4080 | -0.001 | -0.1434 | No |
| 21 | PHYH |  |  | 4372 | -0.006 | -0.1744 | No |
| 22 | GNPAT |  |  | 4548 | -0.010 | -0.1910 | No |
| 23 | PEX13 |  |  | 4933 | -0.017 | -0.2290 | No |
| 24 | HSD17B4 |  |  | 4950 | -0.017 | -0.2248 | No |
| 25 | PFKM |  |  | 5015 | -0.019 | -0.2257 | No |
| 26 | PEX26 |  |  | 5249 | -0.023 | -0.2443 | No |
| 27 | AR |  |  | 5543 | -0.029 | -0.2676 | No |
| 28 | ABCA2 |  |  | 5665 | -0.032 | -0.2703 | No |
| 29 | GCLM |  |  | 5804 | -0.034 | -0.2741 | No |
| 30 | NEDD4 |  |  | 5891 | -0.036 | -0.2715 | No |
| 31 | ABCA3 |  |  | 6089 | -0.040 | -0.2801 | No |
| 32 | GSTK1 |  |  | 6180 | -0.042 | -0.2757 | No |
| 33 | FADS2 |  |  | 6359 | -0.046 | -0.2800 | No |
| 34 | PEX6 |  |  | 6622 | -0.053 | -0.2914 | No |
| 35 | SLC35B2 |  |  | 6978 | -0.064 | -0.3098 | Yes |
| 36 | IDH2 |  |  | 6985 | -0.064 | -0.2881 | Yes |
| 37 | SOD1 |  |  | 7066 | -0.067 | -0.2740 | Yes |
| 38 | PEX16 |  |  | 7068 | -0.067 | -0.2509 | Yes |
| 39 | NPC1 |  |  | 7097 | -0.068 | -0.2306 | Yes |
| 40 | SLC22A18 |  |  | 7123 | -0.069 | -0.2096 | Yes |
| 41 | PEX1 |  |  | 7266 | -0.073 | -0.2004 | Yes |
| 42 | HSD17B11 |  |  | 7363 | -0.077 | -0.1848 | Yes |
| 43 | MLYCD |  |  | 7368 | -0.077 | -0.1585 | Yes |
| 44 | PXMP2 |  |  | 7501 | -0.082 | -0.1453 | Yes |
| 45 | ABCA5 |  |  | 7590 | -0.085 | -0.1256 | Yes |
| 46 | IDI1 |  |  | 7851 | -0.099 | -0.1211 | Yes |
| 47 | LONP2 |  |  | 8041 | -0.110 | -0.1046 | Yes |
| 48 | FADS1 |  |  | 8186 | -0.120 | -0.0792 | Yes |
| 49 | DHCR24 |  |  | 8321 | -0.134 | -0.0478 | Yes |
| 50 | EFHC1 |  |  | 8356 | -0.140 | -0.0030 | Yes |
| 51 | ATXN1 |  |  | 8456 | -0.153 | 0.0389 | Yes |
Table: GSEA details [plain text format]

  

Fig 2: HALLMARK\_BILE\_ACID\_METABOLISM: Random ES distribution      
 Gene set null distribution of ES for **HALLMARK\_BILE\_ACID\_METABOLISM**
